# Nanotechnology for the Treatment of Allergic Conjunctival Diseases

**DOI:** 10.3390/ph13110351

**Published:** 2020-10-29

**Authors:** Yu-Chi Liu, Molly Tzu-Yu Lin, Anthony Herr Cheun Ng, Tina T. Wong, Jodhbir S. Mehta

**Affiliations:** 1 Tissue Engineering and Cell Therapy Group, Singapore Eye Research Institute, Singapore 169856, Singapore; liu.yu.chi@seri.com.sg (Y.-C.L.); molly.lin.t.y@seri.com.sg (M.T.-Y.L.); 2 Department of Cornea and External Eye Disease, Singapore National Eye Centre, Singapore 168751, Singapore; tina.wong.t.l@singhealth.com.sg; 3 Ophthalmology and Visual Sciences Academic Clinical Program, Duke-NUS Medical School, Singapore 169857, Singapore; 4 School of Materials Science and Engineering, Nanyang Technological University, Singapore 639798, Singapore; NGHE@ntu.edu.sg; 5 Ocular Therapeutics and Drug Delivery Group, Singapore Eye Research Institute, Singapore 169856, Singapore; 6 Department of Glaucoma, Singapore National Eye Centre, Singapore 168751, Singapore

**Keywords:** allergic eye diseases, nanomedicine, drug delivery system

## Abstract

Allergic conjunctivitis is one of the most common external eye diseases and the prevalence has been increasing. The mainstay of treatment is topical eye drops. However, low bioavailability, low ocular drug penetration, transient resident time on the ocular surface due to tear turnover, frequent topical applications and dependence on patient compliance, are the main drawbacks associated with topical administration. Nanotechnology-based medicine has emerged to circumvent these limitations, by encapsulating the drugs and preventing them from degradation and therefore providing sustained and controlled release. Using a nanotechnology-based approach to load the drug is particularly useful for the delivery of hydrophobic drugs such as immunomodulatory agents, which are commonly used in allergic conjunctival diseases. In this review, different nanotechnology-based drug delivery systems, including nanoemulsions, liposomes, nanomicelles, nanosuspension, polymeric and lipid nanoparticles, and their potential ophthalmic applications, as well as advantages and disadvantages, are discussed. We also summarize the results of present studies on the loading of immunomodulators or nonsteroidal anti-inflammatory drugs to nano-scaled drug delivery systems. For future potential clinical use, research should focus on the optimization of drug delivery designs that provide adequate and effective doses with safe and satisfactory pharmacokinetic and pharmaco-toxic profiles.

## 1. Allergic Conjunctival Diseases

The conjunctiva is immunologically active as it is constantly exposed to the environment and surrounding allergens. In clinical practice, ocular allergy is a common immunological hypersensitivity disorder and represents one of the most common external eye diseases encountered. Allergic conjunctivitis has been estimated to affect 6–30% of the general population, but up to 30% of children, and this prevalence is expected to increase worldwide [1,2]. Approximately 30% of patients may have recurrent episodes, with intense and persistent symptoms and signs [1], affecting work productivity and thereby having secondary economic effects [2]. The economic impact of ocular allergic diseases is estimated to be over $2 billion US dollars annually in prescriptions generated by primary care physicians and eye care specialists, and this does not include over-the-counter medications projected to be 10-fold more than prescriptions [3].

**Figure 1:**
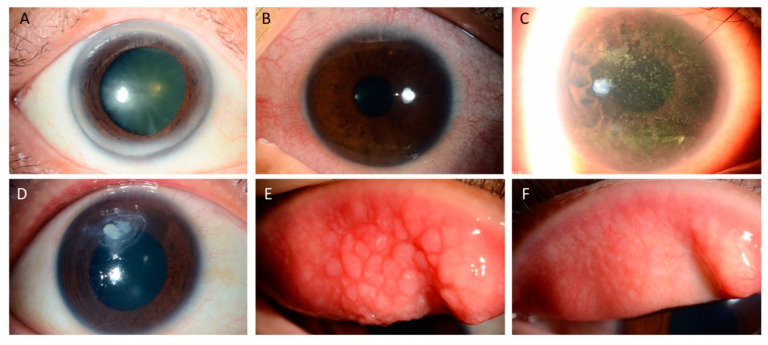
Clinical manifestations of allergic conjunctival diseases. (**A**) Normal conjunctiva and cornea (**B**) Conjunctival hyperemia and pannus (**C**) Corneal punctate epithelial erosions, (**D**) non-infectious shield ulcer, (**E**) Giant papillae of VKC, and (**F**) Residual papillary reaction as well as hypertrophy and resolution of giant papillae in the tarsal conjunctiva after topical medical treatment.

Clinically, allergic conjunctivitis is a generic term that includes seasonal allergic conjunctivitis (SAC), perennial allergic conjunctivitis (PAC), vernal keratoconjunctivitis (VKC), and atopic keratoconjunctivitis (AKC). They all share the same immune-pathophysiology in which a cascade of immunoglobulin (Ig) E-involved Type I allergic reactions, mast cell-initiated immunological responses, and T lymphocyte-mediated type IV hypersensitivity, are triggered by environmental allergens [4,5]. The clinical symptoms and signs vary among these four conditions, but typical presentations, including bilateral itch, photophobia, dryness, eyelid swelling, conjunctival hyperemia, mucous or watery discharge, conjunctival swelling (chemosis), and tarsal conjunctival papillary reaction, occur in all forms (Figure 1). The disease history and ocular presentations for SAC, PAC, VKC, and AKC are summarized in Table 1.

## 2. Treatment of Allergic Conjunctival Diseases

**Figure 2:**
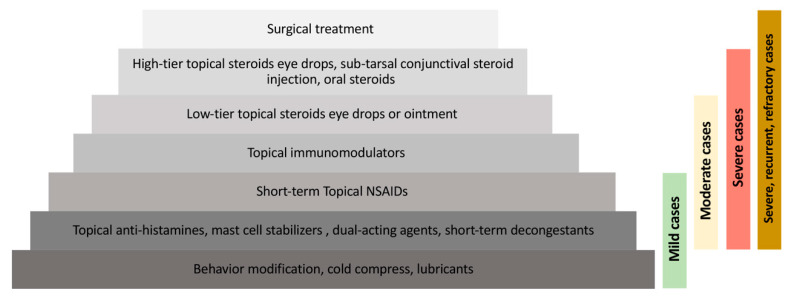
Overview of a stepwise approach to the treatment of allergic conjunctivitis.

A proportion of patients with allergic conjunctival diseases often self-medicate or fail to seek help for their symptoms, and hence allergic conjunctivitis is often underdiagnosed and undertreated [6]. In general, a stepwise approach is adopted by ophthalmologists for the management of ocular allergic diseases (Figure 2).

### 2.1. Supportive Treatment

Behavioural modification including identification and avoidance of the exogenous antigens, such as pollens or animal dander, is the primary management approach, followed by non-pharmacological therapy such as avoidance of eye rubbing and cold compression for symptomatic relief. Artificial tears lubricants, which usually consist of saline solutions combined with a viscosity agent, such as methylcellulose or polyvinyl alcohol, can be supportive treatment, helping to dilute and remove allergens and inflammatory mediators from the ocular surface.

### 2.2. Topical Anti-histamines

For the mild and intermittent cases, topical anti-histamines, such as levocabastine and emedastine (both are selective H1 antagonists), remain the mainstay to quickly relieve the symptoms with the average onset of action of 3 to 15 min, by binding to histamine receptors [7]. However, besides inhibiting histamine release, these drugs have no effect on other mediators involved in the allergic response such as leukotrienes and prostaglandin, and therefore are rarely clinically sufficient as monotherapy. In addition, they relieve symptoms and signs for a short period of time only, necessitating repeated instillations of up to four times per day [8]. The use of combinations of anti-histamine with vasoconstrictor (such as naphazoline-pheniramine) has been shown to be more effective than topical anti-histamine alone [9]. However, it also has short duration of action, and is associated with compensatory chronic vascular dilation after consecutive use for 5–7 days [9,10].

### 2.3. Topical Mast Cell Stabilizers

Mast cell stabilizers, such as sodium cromoglicate and lodoxamide, inhibit the activation, chemotaxis, degranulation and cytotoxicity of neutrophils, eosinophils, monocytes and mast cells and are effective in mild allergic cases [10]. Mast cell stabilizers require long regular dosing for several weeks for loading for the prophylactic effect [9], hence patient compliance may be a potential problem that necessitates the use of sustained drug release systems. Moreover, due to their single-acting effect and the current availability of dual-acting agents (see below), mast cell stabilizers are seldom used as monotherapy.

### 2.4. Topical Dual-Acting Agents

Recently, dual-acting agents that combine anti-histamine and mast-cell stabilizing activity, such as olopatadine, azelastine, epinastine and ketotifen, have become the first-line treatment for mild forms of allergic conjunctivitis because of their superiority in ameliorating allergic symptoms and signs quickly, supported by various clinical studies, compared to either anti-histamines or mast cell stabilizers alone [8]. Their anti-histaminic effect reduces the ocular allergic response such as itching in the early phase, whereas the mast cell-stabilizing properties suppress the release of inflammatory mediators such as cytokines and lipid mediators, which are associated with the late-phase response of allergic conjunctivitis [11,12]. Dual-acting drugs also demonstrate good safety profile even with chronic use [11]. However, they must be used for long durations to be effective, hence long-term compliance can be an issue.

### 2.5. Topical Non-steroidal Anti-inflammatory Drugs

Topical non-steroidal anti-inflammatory drug (NSAID) blocks the cyclooxygenase pathway and inhibits the production of prostaglandins, which plays a role in IgE-mediated allergic reaction [8]. Certain topical NSAIDs have been approved by the US Food and Drug Administration (FDA) for the use in ocular atopy, but their efficacy varies greatly [10]. Although it can provide rapid relief of ocular symptoms, generally it is not commonly prescribed and is used only for short-term due to its adverse effects such as ocular irritation and corneal melting for long-term use [13]. For those patients whose clinical manifestations are inadequately controlled in spite of the use of dual-acting agents or for those who have a contraindication for the use of topical steroids, a topical NSAID may provide short-term benefits.

### 2.6. Corticosteroids

For moderate and severe allergic conjunctivitis or when conservative treatment fails, topical corticosteroids are potent and effective treatments by inhibiting a board range of inflammatory cascades in the allergic responses [10]. However, it is well known that long-term use of corticosteroids is associated with a wide range of adverse effects including intraocular pressure elevation, cataract formation, delayed wound healing and increased susceptibility or exacerbation of underlying infection [14,15]. Hence, its use should be short-term, judicious, carefully monitored and reserved for exacerbations, that result in moderate to severe discomfort and/or decreased vision. For cooperative patients, sub-tarsal injection of short-acting corticosteroids such as dexamethasone phosphate (4 mg/mL) or longer-acting corticosteroids such as triamcinolone acetonide (40 mg/mL) can be used as an alternative to topical eye drops [10]. However, monitoring of intraocular pressure is mandatory.

### 2.7. Topical Immunomodulators

Immunomodulators, such as cyclosporin A and tacrolimus, suppress T-helper cell-mediated response, B-cell proliferation, and prostaglandin and cytokine release, especially interleukin (IL)-2, IL-4, and IL-5, which play roles in ocular allergic diseases [16]. It also blocks the release of histamine from mast cells. The potent anti-inflammatory effects and favorable side effect profiles enable it as an efficacious alternative to topical steroids to control the diseases, especially in refractory cases [17]. Tacrolimus and cyclosporin A have similar functional mechanisms, but the former present 50–100 folds higher potency [18]. Both drugs are hydrophobic and have a high molecular weight, which could allow greater permeation in the conjunctiva than in the cornea, as the conjunctiva is up to 20 times more permeable to lipophilic and high-molecular-weight drugs than is the cornea [19]. Several clinical trials have demonstrated that topical tacrolimus, in the form of either ointment or suspension, significantly improved the clinical symptoms and signs in patients with AKC or VKC, in a concentration range of 0.005% to 0.1%, treatment frequency once to four times daily, and treatment duration of 1 to 29 months [20]. Early medical treatment with topical tacrolimus also prevents the development of serious ocular complications of VKC, such as shield ulcers or limbal stem cell deficiency [20].

However, tacrolimus has a water solubility of only about 1 µg/mL, and is also susceptible to hydrolysis that leads to very low stability in aqueous solutions [21]. Attempts have been made to prepare ophthalmic tacrolimus in castor oil, olive oil and dextrin [22]. These preparations, nevertheless, may be associated with several side effects such as ocular irritation, redness, burning and itching sensation [22]. Nanomedicine therefore becomes a potential alternative solution to deliver these drugs. As allergic conjunctival diseases are chronic and recurrent conditions, long-term use of topical eye drops and good patient compliance are necessary [16]. However, topical eye drops have a poor bioavailability of less than 10% and a short duration of action and therefore frequent application is required to achieve a therapeutic level [23,24]. To overcome these limitations, several drug delivery platforms have been introduced and developed, including nanomedicine-based ocular delivery.

### 2.8. Surgical Management

For those patients with VKC and cobblestone papillae (Figure 1) refractory to medical treatment, surgical excision of the giant papillae combined with mitomycin C, amniotic membrane grafts or conjunctival autografts, has been reported to reduce the corneal complications [25]. However, these surgical options serve as the last-line treatment and are not routinely performed in all severe cases.

## 3. Introduction of Nanotechnology

The underlying remarkable value of the nanotechnology-based approach is attributed by its unique characteristic of nanoscale. The reduction of size per unit volume ratio enhances the surface area of materials, empowering smaller sized particles like nanocarriers to improve corneal permeation, higher loading and release efficiency of drugs with a relatively lower clearance rate [26]. In comparison to other drug delivery systems, the nanotechnology-based approach is superior in the way that its formations can be adjusted according to the solubility of drug of interest. It has been documented to be particularly effective for loading drugs that exhibit poor solubility in aqueous solution, like lornoxicam, cyclosporin-A, and tacrolimus [27,28]. The dosage forms such as nanomicelles and nanoemulsions enable the entrapment of hydrophobic drugs in the lipophilic inner core while the hydrophilic outer layer facilitates its delivery in aqueous environments of the human body [29]. Hence, the encapsulation of the drugs not only serves as a shield in preventing undesirable drug release, but also governs the potential of sustained-release of drugs with extended retention time on the ocular tissues, achieving higher therapeutic efficacy [30]. In addition, by employing an appropriate formulation as carriers like liposomes, the local toxicity or irritation associated with free drugs can be reduced [31]. The nanocarriers can be also tailored with appropriate ligands, antibodies, or polymers to enhance its ability to cross biological membranes such as corneal and conjunctival epithelium [32]. Besides this, it assists in site-specific drug localization and enhances drug retention time for its therapeutic effects. For ocular drug delivery, instilling particulate formulated by liposomes, nanoparticles, and lipid emulsions are often eliminated rapidly from the tear fluid [33]. Luckily, the flexibility of modifying nanocarriers with mucoadhesive property enables the interaction between drug-loaded nanocarriers and mucin present on the ocular surface, improving ocular bioavailability with prolonged residence time in the ocular surface [31]. Incorporating mucoadhesive polymers such as carbopol, hyaluronic acid, sodium carboxymethylcellulose, and xanthan gum enhances the viscosity of dosage, facilitating sustained and controlled release of drugs [34].

In ophthalmic practice, topical eye drops, subconjunctival or intracameral injections have been considered as conventional routes to administer active pharmaceutical ingredients for anterior segment diseases. However, common drawbacks such as low drug penetration and bioavailability across ocular surface barriers, the transient residence time at the targeted site, the dependence of patient compliance and tolerance, as well as potential side effects resulting from frequent administrations [35]. Hence, the adoption of nanotechnology provides a promising strategy to optimize ophthalmic drug delivery. Such a paradigm shift is possible due to their flexibility to design, synthesize, and modify the shape, size and surface properties of the nano-scaled materials, to optimize the ocular penetration, bioavailability, and drug specificity, and to prolong drug retention time, thereby achieving sustained delivery and controlled release of therapeutic drugs for ophthalmic treatments [31,36,37].

## 4. Nanotechnology-Based Drug Delivery Systems for Ocular Disorders

**Figure 3:**
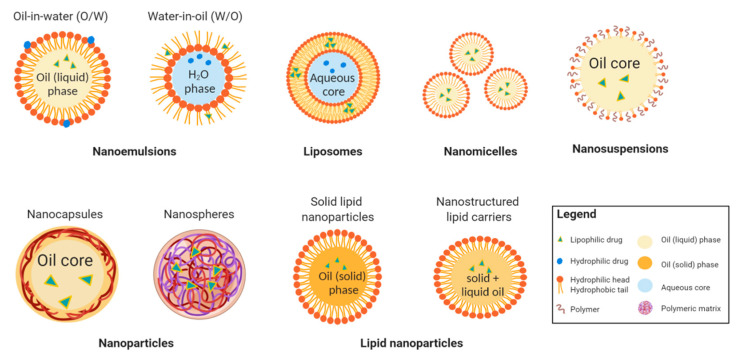
Schematic representation of nanotechnology-based drug delivery systems for ocular diseases (created with BioRender.com).

In the era of nanotechnology, colloidal carrier systems such as nanoemulsions, liposomes, nanomicelles, nanosuspension, polymeric and lipid nanoparticles are highly promising vehicles for ocular drug delivery (Figure 3). These drug forms utilize the disperse systems to incorporate drugs of interest into the individual formulation for controlled release and site-specific drug delivery [38]. Inorganic nanocarriers, such as gold, mesoporous silica, and magnetic iron oxide nanocarriers, have also been proposed [39]. Typical gold nanocarriers have an inert gold core and an active outer that conjugates with targeted drugs [40]. The porous property of mesoporous silica allows a high surface area and high pore volume to absorb and encapsulate molecules [41]. For magnetic iron nanoparticles, by applying external magnetic field, the loaded drug can be concentrated in the targeted tissue. However, the magnetic gradient can not be concentrated in three dimensions, and the particles’ direction can not be kept once the magnetic field is removed from outside [39]. Moreover, these inorganic nanocarriers are not biodegradable [39], posing a potential issue in biological toxicity. To date, there have been no documented studies reporting their applications in the treatment of allergic conjunctival disease.

### 4.1. Nanoemulsions

Nanoemulsions are spontaneous biphasic dispersion of two immiscible liquids, stabilized by surfactant. The droplets can be easily produced through water-in-oil (W/O) or oil-in-water (O/W) emulsification. In ocular drug delivery, nanoemulsion (O/W) formulation is favoured as it allows encapsulation of immunosuppressive drugs like cyclosporin A and tacrolimus which have low solubility in the aqueous solvent [42]. The presence of surfactant allows nanoemulsions thermodynamic stability and enhances membrane permeability for drug uptake into the deeper layers of the eye [43,44]. In addition, it can help stabilize the tear film by restoring the lipid and water component, while the use of emulsifier is known to improve the wettability of the tear film [45]. However, the application of high surfactant concentration to maintain the stability of formulating hydrophobic drugs may lead to visual blurring, ocular burning, conjunctival hyperemia and ocular intolerance [46].

### 4.2. Liposomes

Liposomes are established formulations that have been widely applied in facilitating drug delivery since their first discovery in the 1960s [47]. Liposomes are spherical vesicles formed by one or more natural phospholipid bilayers enclosing in an aqueous inner core [44,48]. Owing to their small size, biocompatibility, and the unique ability to entrap both hydrophilic drugs at the aqueous inner phase and hydrophobic molecules within the vesicle bilayer membrane, liposomes represent promising candidates for ophthalmic drug delivery [16]. Over the years, liposomes have been shown by numerous studies to generate effective ocular drug delivery systems for both anterior and posterior segment diseases [44]. Their gradual drug release profile with a less-extent of initial burst at an early stage of administration has gained popularity in ocular applications with great potential in improving the interaction between the liposomal drugs and ocular site of action for an extended period of time. The application of liposomes as drug carriers is determined by their properties such as lipid composition, surface charge, and the preparation methods [44]. Positively-charged liposomes have the capacity to increase drug encapsulation efficiency and to enhance binding affinity with the corneal surface by capturing the negatively-charged sialic acid in mucin on ocular surface [31], allowing better corneal penetration of the drug and assisting drug transfer from liposomal carriers to epithelial cell membranes [49]. A study on flurbiprofen loading has shown that the loading efficacy was 1.5 times higher when adopting deformable liposomes with 0.05% chitosan compared to loading with conventional liposomes (90.2% versus 63.7%) [31].

### 4.3. Nanomicelles

Nanomicelles are commonly used to formulate lipophilic therapeutic compounds for drug delivery purposes. It can be easily prepared by dispersing amphoteric molecules into clear aqueous solutions to form a vesicular lipid monolayer with an enclosed hydrophobic core and hydrophilic corona [50]. Similar to the emulsion and liposomal carrier systems, nanomicelles are extremely small in size and made of amphiphilic molecules. Its great drug loading efficiency and nanomicellar formulations have contributed greatly in improving ocular bioavailability [51]. Polyethylene glycol (PEG) is often added when formulating drugs in nanomicelles due to their ability to improve the stability of nanomicelles in physiological environments and precorneal fluid [50,52]. Nanomicelles have been shown to be well-tolerated in human corneal epithelial cells with no cytotoxicity observed [50]. An in-vivo study of dexamethasone-loaded nanomicelle using copolymers of polyhydroxyethylaspartamide (PHEAC(16)) and PEGylated PHEAC(16) for anterior segment delivery has shown a better bioavailability compared to its suspension [53], showing an alternate platform to deliver drugs.

### 4.4. Nanoparticles

Nanoparticles are another frequently applied colloidal carriers which have been employed efficiently as ocular drug delivery systems for the past decades. Commonly used materials include lipids, proteins, and biodegradable polymers derived either synthetically from poly (lactide-co-glycolide) (PLGA), polylactic acid (PLA), polycaprolactone (PCL), or naturally from albumin, gelatin, sodium alginate, and chitosan [54]. In this formulation, a drug can be loaded in or adsorbed onto the surface of nanocapsules or nanospheres (Figure 3) [54]. Nanocapsules are vascular systems in which a drug can be dissolved in the hydrophobic inner core enclosed by the polymeric envelop whereas in nanospheres, the drug is evenly dispersed within the polymer matrix [54,55]. Upon administration onto the delivery site, the therapeutic active substances can be released through diffusion, enzymatic reaction, polymer degradation, or ion exchange mechanisms in a controllable fashion [43]. Many investigations have been made in modifying the surface characteristics of the nanoparticles. Positive-charged mucoadhesive polymers on the surface of nanoparticles serve as a crucial factor in influencing the duration of residence time in the ocular surfaces, as well as the degree of penetration for drug disposal [56]. The PLGA-nanoparticles formulation has been successfully developed with high drug entrapment efficiency of greater than 85% and presents a sustained drug release profile compared to the conventional eye drops [56], proving its capability of being a promising candidate in carrying drugs to the ocular site of interest with therapeutic efficacy. The pitfalls of loading drugs in this system include difficult production, stability issues during storage, aggregation of particles and possible systemic toxic effects from polymer degradation products which should be carefully investigated [46,57].

### 4.5. Lipid Nanoparticles

Recently, lipid nanoparticles have been developed based on the principle of oil-in-water emulsion [33], and are deemed as a superior method over nanoemulsion, liposome, and nanoparticles due to their low in-vivo toxicity, great long-term stability, simple scale-up production, and possibility to undergo sterilization [46]. In general, lipid nanoparticles can be classified into two categories: solid lipid nanoparticles and nanostructured lipid carriers (Figure 3). The distinct feature of solid lipid nanoparticles being encapsulating lipophilic drugs is its solid lipid core which retards the drug mobility, achieving a better-controlled drug release profile and higher drug bioavailability when compared to nanoemulsion formulations which utilize liquid lipid for drug incorporation [58]. Moreover, the ocular drugs delivered using solid lipid nanoparticles system has been reported to have a longer residence time on the ocular surface and conjunctival sac than using an aqueous eye drops [59].

Nanostructured lipid carriers were developed to address the drawbacks of solid lipid nanoparticles, including the limited drug loading capacity and expulsion of loaded drugs during storage [46]. By mixing incompatible liquid lipid with solid lipid, the disorganized crystalline structure of the lipid is formed [46]. Such nanostructured lipid carriers result in a larger distance between the fatty acid chains of the lipid core, expanding the drug loading capacity [46]. The production of nanostructured lipid carriers is especially beneficial for drugs with higher solubility in liquid oils [60]. The mixture of a larger amount of liquid lipids with lesser solid lipids allows the formation of nanosized liquid lipid droplets within the solid core, enabling drug protection from fast degradation while prompting prolonged drug release, and it can even prevent the expulsion of highly lipophilic drugs during cooling or storage process [61]. The crystalline-contributed drug expulsion in solid lipid nanoparticles formulation can also be lifted by making “structureless nanostructured lipid carriers” which employs the mixture of a particular liquid and solid lipids that solidify without forming crystalline upon cooling [60]. It has also been shown that nanostructured lipid carriers improve drug protection and entrapment efficiency than the normal solid lipid nanoparticles [46,62].

### 4.6. Nanosuspensions

Nanosuspension is an emerging technology that suspends poorly soluble or poorly permeable drugs in an appropriate dispersion medium. The preparation of nanosuspensions adopts “bottom-up technology” or “top-down technology” [63]. To form nanosized particles, the former technology uses an integrating method like precipitation, microemulsion, and melt emulsification techniques while the latter involves disintegrating larger particles into nanoparticles by means like high-pressure homogenization and milling [63]. The distinct nature of nanosuspension can not only circumvent high osmolarity produced by ophthalmic solutions, but also resolve saturation- and solubility-associated issues of hydrophobic drugs in tear fluids while keeping drugs in the cul-de-sac for a longer period of time with sustained drug release [64]. Studies have shown that glucocorticoids such as prednisolone, dexamethasone, and hydrocortisone formulated in nanosuspensions using high-pressure homogenization had more intense therapeutic effects and higher drug absorption compared to free solutions and microcrystalline suspensions of the drugs [65]. In addition, drugs prepared in nanosuspension with PLGA were observed with improved precorneal retention time and ocular permeation. Drugs encapsulated in lyophilized nanosuspension also showed higher stability than conventional formulations [66].

### 4.7. Advantages, Disadvantages and Challenges of Nanotechnology Based Drug Delivery Systems

The substantial improvement in in-vivo trans-corneal permeability and drug retention time by lipid-based nano-formulations over conventional formulations has propelled nanomedicine a step forward to its great contribution in ocular drug delivery [62]. Without doubt, the emergence of interdisciplinary principles involving nanotechnologies has brought revolutionary impact in ophthalmic drug delivery systems. Ocular drug-loaded nanomedicine establishes superiority over conventional eye drops with greater bioavailability, higher therapeutic efficacy, sustained and controlled release drug profile. With the help of nano-formulation as protective shield for therapeutic agents, the drug-associated ocular irritation and local toxicity at higher drug concentrations can be reduced [67]. By choosing among a series of developed nanocarriers, along with the biodegradable and biocompatible biopolymers with mucoadhesive properties, as well as suitable excipients, constituents can be combined in different ratios to optimize the therapeutic efficacy of the tailored nanomedicine to meet specific clinical needs [30,68]. However, there are also drawbacks. For example, positively-charged biopolymers containing nano-formulation prolong the drug retention time at the ocular surface. Prolonged residence time might also potentially provoke local toxicity that warrants more investigations. Besides, corneal damage could occur when a too high concentration of surfactants is present in the formulation [69]. The choice of surfactants therefore plays a pivotal role in avoiding ocular irritation while maintaining the stability of the formulation. The presence of surfactants in some cases may cause a sticky sensation and blurred vision upon instillation, hence impeding patient compliance [70]. One associated challenge across all nano-formulations lies in its concerns with different toxicity profiles when applying different excipients and polymers. Several discussions have also elucidated the potential antigenicity and thrombogenicity due to nanoparticles’ properties, such as size or surface characteristics [71].

The clinical translation of nanomedicine technology is usually considered more complex, time-consuming and costly compared to conventional drug formulation technology [72]. Biological challenges are the main hurdles. Animal models reflect only a narrow spectrum of the clinical disease, and the differences in the anatomy and physiology of animals may pose challenges in formulations. Moreover, similar potential toxic effects from storage instability, if left unresolved, could pose physiological adverse effects after administration. It can also be hard to control homogenous particle size in nanoparticle dosage formulation [73]. Quality control, including consistent product yield, purity of the product, and good reproducibility among batches, has to be ensured and evaluated comprehensively prior to clinical applications. In addition, not all nano-formulations can undergo aseptic productions via autoclaving [46], which may pose concerns for clinical applications. For some nano-formulations, it may be difficult to produce in a large scale with Good Manufacturing Practice (GMP) standards. [69] The lack of uniform standard in regulatory approval examinations may also hamper the regulatory approval processes [71]. The unique customizable feature of each nano-formulation may make a lack of standardized protocols such as in-vivo tolerance tests [74]. There is a need for regulatory standards for validated and sensitive, and also for standard protocols comprising in-vitro, ex-vivo and in-vivo experiments to adequately evaluate the data of pre-clinical and early-phase clinical trials. All these contribute to the slow pace of the clinical translation of nanomedicine. However, these challenges for the application of nanomedicine in ophthalmology may be less in comparison with those in systemic applications, as the route of administration and targeted tissue are more localized.

Hence, it is important to carefully leverage the strengths and limitations of each dosage formulation to maximize the therapeutic effects of active pharmaceutical agents over an extended period of time while minimizing the potential systemic or local toxic side effects. Continuous optimization with appropriate modification and functionalization of formulation, such as polymer properties and surface characteristics, would serve a valuable support for clinical translation. The advantages and disadvantages of nanomedicines in ocular applications are summarized in Table 2.

## 5. Nanotechnology for the Treatment of Allergic Conjunctival Diseases

Recent pre-clinical studies have demonstrated the safety and efficacy of the applications of nanotechnology to deliver immunomodulators, NSAIDs and corticosteroids, three effective treatment options for allergic conjunctivitis. In general, there are two strategies for a novel drug delivery system to enhance the bioavailability: increasing the drug penetration through the ocular surface and prolonging residence time of drug on the ocular surface.

### 5.1. Immunomodulatory Agents

Cationic nanoemulsions loaded with cyclosporin A have been investigated and developed for the past decade and are an example of successful application of bench work to clinical trials. With the addition of bio-adhesive substances (e.g., cationic nanoemulsions) to the nano-system, the drug can be more efficiently delivered at appropriate concentrations, with the use of bio-adhesiveness as an electrostatic interaction to prolong the residence time of the drug on the ocular surface, as the positively-charged nano-droplets are attracted to the negatively-charged cell membranes [75]. In a phase 3, multicenter, double-masked, vehicle-controlled clinical trial with 169 pediatric patients with active and severe VKC, treatment with cyclosporin A cationic emulsions significantly improved patients’ symptoms, signs and quality of life after 4 months’ course of treatment in both high-dose (four times daily) and low-dose (twice daily) groups compared to the vehicle group [42].

Topical tacrolimus nanoemulsion formulation has also been proposed. In a pharmacokinetic study in rabbits, tacrolimus nanoemulsions administrated topically demonstrated four-fold ocular bioavailability compared to conventional eye drops because of faster movement of nano-sized globules through paracellular or transcellular junctions [68]. In addition, encapsulation of tacrolimus into the inner oily phase of nanoemulsions decreased the local toxicity and ocular irritation associated with drugs, thereby improving the safety profile [68]. Nanoemulsions have a viscosity similar to tacrolimus eye drops but do not cause blurring of vision due to their nano-size [68]. Of note, most of the oils used for the preparation of nanoemulsions possess some anti-inflammatory properties which may synergize with tacrolimus [76].

Tacrolimus-loaded PLGA-nanoparticles has also been developed [77]. PLGA has been approved by FDA for ocular use due to its biocompatibility and good biodegradability, and PLGA-nanoparticle systems have been considered promising to deliver drugs in a sustained and controlled manner [78]. Alshamsan et al. optimized the formulations of tacrolimus-loaded PLGA-nanoparticles in terms of the characterization parameters, trans-corneal permeation and stability [77]. The mean particle size and its distribution, polydispersity, zeta-potentials, morphology, drug encapsulation and loading capacity remained unchanged after 1-month storage at 25 °C. In a rabbit model, PLGA-nanoparticles improved corneal, conjunctival and aqueous humor bioavailability of tacrolimus. A considerably higher tacrolimus concentration was detected in ocular tissues even at 24 h after the instillation compared to the that of conventional eye drops [77]. There were no obvious adverse effects observed clinically in corneas, conjunctiva and iris.

Compared to polymeric nanoparticles, lipid nanoparticles can be prepared using techniques that are easier to scale up and are stable during storage [58]. Loading cyclosporin A to lipid nanoparticles has been evaluated in rabbit eyes. With the presence of lipase/co-lipase enzyme complex, the drug release was found to be enzyme-dependent. High loading efficiency up to 96%, good physical stability to avoid aggregation, and improved penetration of drug across the cornea tissue was observed [79], suggesting its potential to be an alternative treatment option.

**Figure 4:**
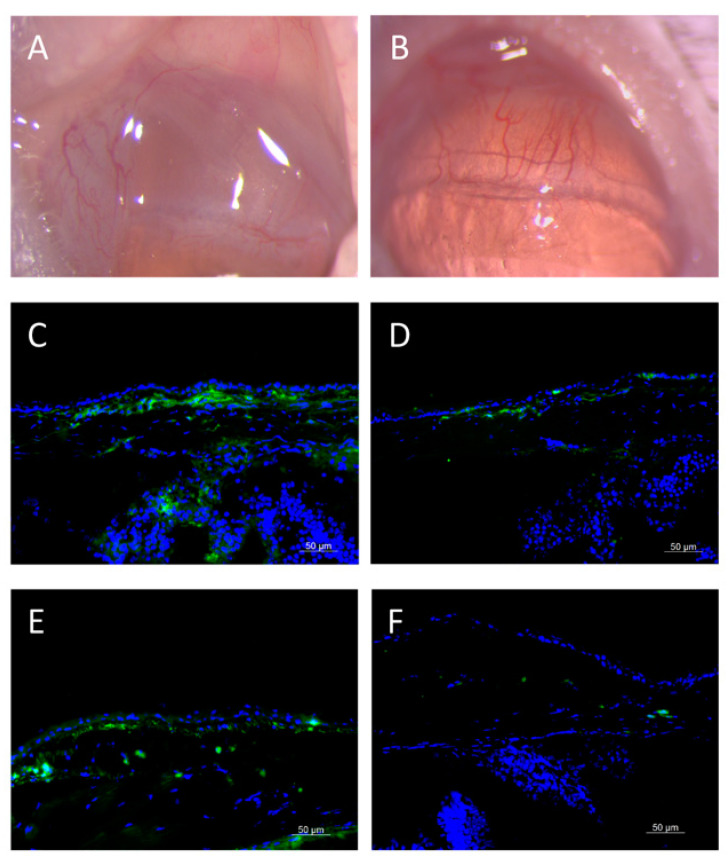
Effects of single-dose subconjunctival injection of liposomal tacrolimus for the treatment of allergic conjunctivitis in a mouse model. (**A**) On slit-lamp evaluation, mice with the induction of allergic conjunctivitis presented marked chemosis and congestion of conjunctival vessels, and (**B**) liposomal tacrolimus treatment reduced these signs. (**C**–**F**) On the immunohistochemistry staining, mice with allergic conjunctivitis exhibited significant expression of (**C**) IL-4 and (**E**) CD4, two cell markers for allergic reaction. (**D**,**F**) The expression of IL-4 and CD4 was less in the liposomal tacrolimus group, respectively.

Recently, our group encapsulated tacrolimus in 1-palmitoyl-2-oleoyl-sn-glycero-3-phosphocholine (POPC) liposomes using the thin-film hydration method. In a rabbit model, we observed sustained release of tacrolimus for 6 weeks, and the drug concentration in the conjunctiva was higher than that in the conventional eye drops. A single subconjunctival injection of liposomal tacrolimus effectively suppressed the chemosis and congestion of conjunctival vessels, and significantly reduced the expression of IL-4 and CD4 T cells in the conjunctiva in a mice allergic conjunctivitis model (Figure 4; data not published).

Modification of liposomes with edge activators such as propylene glycol (PG) or other surfactants could decrease the vesicle aggregation and increase the liposomal elasticity and therefore enhance the trans-ocular permeation [67]. This is especially useful when loading hydrophobic, high molecular weight molecules such as tacrolimus. Garg et al. demonstrated that PG modified liposomes had 5-fold and 13-fold higher corneal permeation than conventional liposomes and tacrolimus eye drops, respectively, in a rabbit model [67]. The retention time in corneal tissue was also significantly prolonged, providing a novel method to deliver drugs more effectively. Table 3 summarizes the literature on the use of nanotechnology-based drug delivery systems for immunomodulatory agents for the treatment of allergic conjunctival diseases.

### 5.2. NSAIDs

Biodegradable polymeric PLGA nanoparticles encapsulating dexibuprofen has been proposed. A corneal membrane model mimicking the lipid structure of the corneal surface was used, and the interactions between the drug delivery system and the ocular surface were studied. The authors confirmed that the use of lutrol as a surfactant in the formulation process produced the best therapeutic efficacy of the NSAIDs in term of its inhibitory effect on ocular surface inflammation. It also exhibits an absence of any irritating ocular phenomena even in high concentrations [56]. In a study reporting flurbiprofen-loaded PLGA nanoparticles, the formulations yielded good entrapment efficacy at 95% as well as continuous and controlled release. It also reduced the ocular surface inflammation scores in a rabbit chemical injury model [80].

Chitosan-based nanoemulsions for ocular delivery of indomethacin was proposed to increase the residence time of the drug in the precorneal area and to provide tissues with long-term drug levels, by utilizing the mucoadhesive and penetration-enhancing properties of Chitosan [81]. The system achieved the therapeutic concentrations in the conjunctiva and aqueous humor, and the levels were significantly higher than those obtained following instillation of topical indomethacin solution.

Muller-Goymann et al. modified the surface and crystal characteristics of lipid nanoparticles with phospholipids and loaded it with diclofenac sodium. The authors showed that the encapsulation efficiency was high and sustained release of diclofenac sodium as well as high permeation through the bio-engineered cornea were achieved [82].

Nanostructured lipid carriers are a newer generation of lipid nanoparticles and can improve drug loading capacity and drug expulsion during storage of lipid nanoparticles, by using structured lipid matrices and surface modification of the particles [83]. Ibuprofen nanostructured lipid carriers have been shown to display controlled-release property in a rabbit model [84]. The permeability coefficients were 1.28 to 1.36 times more than that of the Ibuprofen eye drops, and the area under the curve (AUC) for aqueous humor pharmacokinetics parameters was 3.99 times more than that of Ibuprofen eye drops [84]. Souto et al. optimized nanostructured lipid carriers formulations for the encapsulation of flurbiprofen and showed good in-vitro physico-chemical stability as well as satisfactory results in rabbit in-vivo ocular irritancy tests [85].

Fabrication with nanomicelles is another technique that has attracted attention. Hydrophobic drugs are encapsulated and solubilized into the hydrophobic cores of nanomicelles through hydrophobic interactions. For example, Lornoxicam was incorporated into nanomicelles, and the solubility of the drug was increased 73-fold after encapsulation in the optimum formulation with about 60% of the drug being released within 6 h in rabbits [28]. Studies on loading NSAIDs in nanotechnology-based drug delivery systems are summarized in Table 4.

### 5.3. Corticosteroids

Several studies have reported the successful fabrications of steroid-loaded nano-formulations, such as PEG liposomal prednisolone phosphate, PEG liposomal acetonide phosphate, or triamcinolone acetonide-loaded methoxypoly-PEG-PLGA [88,89,90]. These delivery systems achieved sustained and controlled release over weeks and effectively suppressed the inflammation in experimental uveitis models. The localization of steroid-loaded liposomes in inflamed ocular tissue was also confirmed by histology and immunostaining [88]. However, in the management of allergic conjunctivitis, corticosteroids should be reserved only for moderate to severe cases or for disease exacerbations, with only short-course or intermittent (pulse) doses, such as topical eye drops application or sub-tarsal injections, because of the likelihood that patients develop corticosteroid-related complications from long-term administration [10]. The development of corticosteroid-based nanomedicine should target short-acting formulations that are reversible and with the release profile not beyond a few days.

## 6. Conclusions and Future Directions

Nanotechnology opens a new avenue for the treatment of ocular diseases, especially for insoluble drug molecules. By fabricating drug-filled nanomedicine systems, it has the potential to reduce the degradation, increase the permeability and bioavailability, and prolong the retention time by tailoring the release profiles or by protecting against enzyme activity, thereby achieving sustained drug release and targeted therapeutic concentrations, which have been shown in in-vitro or animal studies. Although there is a distinct advantage over conventional eye drops, all the current approaches are still limited to pre-clinical studies with several challenges that are needed to be overcome, e.g., large-scale manufacturing, before late phase clinical trials are possible. Future work on the design of nanoscale drug delivery should focus on how to obtain satisfactory bioavailability, sustainable release and dose accuracy and at the same time not induce cellular or tissue toxicity. After administration, the influence of the particle size, surface charge, and composition and aggregation on the pharmacokinetic and pharmaco-toxic profiles need to be determined. Finally, clinical studies would be warranted to ascertain the optimal dosing regime for nanotechnology delivering a sustained therapeutic effect.

## Figures and Tables

**Table 1:** Clinical presentation of different types of allergic conjunctivitis.

Type	History	Clinical Presentations
SAC/PAC	Seasonally recur in the presence of allergen Often suffer from other atopic conditions, such as allergic rhinitis or asthma	Bilateral itching, eyelid swelling, conjunctival hyperemia, chemosis and mucoid discharge Intense itching is a hallmark symptom
VKC	Seasonally recur but may persist year-round in tropical climates Predominantly in male children A personal or family history of atopy	Bilateral itching, blepharospasm, photophobia and copious mucoid discharge 2 forms: palpebral and limbal VKC; may present both Palpebral VKC: Diffuse papillary hypertrophy, conjunctival hyperemia, chemosis, giant papillae may develop Limbal VKC: Scattered opalescent mounds at limbus, vascular injection, Horner-Trantas dots Corneal punctate epithelial erosions Pannus Noninfectious epithelial ulcer (shield ulcer)
AKC	Frequently year-round disease with minimal seasonal exacerbation History of atopic dermatitis	Small or medium sized papillae Milky conjunctival edema with variable subepithelial fibrosis is often present Corneal findings such as punctate erosions or epithelial defect may be present

**Table 2:** Advantages and disadvantages of nanotechnology-based ocular drug delivery systems.

**Advantages**
Sustained release in a controlled manner
Improve bioavailability, solubility and penetration of lipophilic molecules into different layers of the eye
Options to employ low-cost, biocompatible, and biodegradable biopolymers in drug encapsulation
Flexibility of modifying nanotechnology-based dosage form (i.e., surface charge, types and ratio of surfactants, polymer properties) to allow better drug permeation and corneal retention time
Choices to tailor nano-formulations based on the solubility of the drugs to achieve optimal therapeutic effects
Nano-formulation can control drug activity by releasing only at the desirable ocular site with prolonged therapeutic effects to reduce frequent doses, improving patient compliance
Encapsulation technique reduces drug-associated ocular irritation and toxicity at higher drug concentration
Great drug efficacy in improving different ocular therapeutics
**Disadvantages**
Nanoparticles can be antigenic that may lead to toxicities and side effects due to its properties (i.e., size, surface characteristics, charge, and hydrophobicity)
Lack of standardized protocol for the in vivo tolerance test
The immunotoxicity in animal models may be unable to accurately predict the safety of nanomedicines in human
The presence of surfactants in some cases could cause sticky feel and blurred vision of the eye upon instillation, impeding patient compliance
Different toxicity profile when employing different excipients and polymers
Difficult to produce in large scale in some nano-formulations and hard to control homogenous particle sizes in nanoparticle dosage formulations
Storage instability (e.g., particle aggregation, active agent expulsion) could lead to possible systemic toxicity
Not all nano-formulations can undergo aseptic productions via autoclaving, which pose concerns in its clinical applications
Absence of standards in regulatory approval examinations due to unique nano-formulations
Biomaterials with longer persistence in particular tissues require stringent evaluation from regulatory agency

**Table 3:** Immunomodulatory agents using nanotechnology-based drug delivery systems for allergic conjunctivitis.

Experimental Models	Drug	Findings
Excised rabbit corneas [77]	Tacrolimus loaded PLGA nanoparticles (PLGA-NPs) via topical eye drops	Enhanced transcorneal uptake of tacrolimus. The t_1/2_ of tacrolimus carried by PLGA-NPs was 1.77-fold greater than conventional eye drops while more than two-fold higher of AUC_0-inf_, AUMC_0-inf_ and MRT_0-inf_ were detected in aqueous humor samples. Effective concentration in cornea and conjunctiva was able to be maintained at 24 h after topical instillation. No adverse effects observed clinically in corneas, conjunctiva, and iris.
Ex-vivo goat eyes for transcorneal permeation study; Rabbits for precorneal retention study [67]	Proglycosomes modified liposomal tacrolimus topical eye drops	5-fold and 13-fold corneal permeation than conventional liposomes and tacrolimus eye drops, respectively. Incorporation of proglycosomes enhanced the drug encapsulation, decreased the vesicle aggregation and increased the liposomal elasticity, thereby enhancing the transocular permeation. Tear samples exhibited prolonged precorneal retention for up to 8 h, with improved intraocular drug levels which exceeded therapeutic levels.
Rabbit corneal epithelial cells for in-vitro study Rabbit corneas for ex-vivo study [79]	Cyclosporin (CsA)-loaded solid lipid nanoparticles	High encapsulation efficiency at 95.6%. The CsA release was lipase/co-lipase complex dependent. The solid lipid nanoparticles improved penetration of CsA across the excised rabbit corneas.
Phase III, multicenter, randomized, double-masked, vehicle-controlled trial [42]	CsA cationic nanoemulsion eye drops	CsA cationic emulsions significantly improved patients’ symptoms, signs and quality of life after 4 months’ course of treatment in both high-dose (four times daily) and low-dose (twice daily) groups compared to vehicle group.
In-vitro study in human corneal epithelial cells; in vivo study in rabbits [37]	CsA-loaded mPEG-PLA nanomicelles via topical instillation	High encapsulation efficiency of CsA nanomicelles of 98.51% with an average particle size of 57 nm. The stability was greatly influenced by the light and temperature. Initial quick drug release of about 73% for 36 h, followed by a prolonged release up to 92 h, achieving total drug release close to 89%. No inflammatory response on cornea, conjunctiva, sclera or iris, but significant in vitro cytotoxicity was present after 24 h of incubation with corneal epithelial cells.

Abbreviation: mPEG-PLA: lyophilized methoxy poly(ethylene glycol)-poly(lactide).

**Table 4:** Encapsulation of NSAIDs using nanotechnology-based drug delivery systems.

Drugs	Experimental Models	Administration	Findings
Dexibuprofen	In-vitro and in-vivo ocular irritation assay on chorioallantoic membrane and in rabbits, respectively. Ex-vivo ocular permeation study in rabbits [56]	Polymeric PLGA nanoparticles (NPs) topical eye drops	In vitro: initial fast release for 150 min, followed by a sustained release for > 24 h. Polyethylene glycol (PEG) increased the interaction between Dexibuprofen-NPs and customized corneal membrane. Using surfactant lutrol to prepare 15% PEG-PLGA-NPs formulation produced the best therapeutic efficacy.
Diclofenac sodium	Bio-engineered human cornea construct [82]	Solid lipid nanoparticles (SLNs)	Phospholipid modified SLNs enabled a higher drug encapsulation efficiency (>90%) with sustained release of diclofenac sodium and high permeation through the cornea construct compared to that formulated without phospholipid.
Ibuprofen	Excised rabbit corneas for in-vitro drug release; rabbits for ocular irritation [84]	Nanostructured lipid carriers (NLCs) topical eye drops	The optimised NLC formulation enabled 95.2% drug entrapment efficiency. Ibuprofen was detected at 6 h after administration comparing to that with eye drops in which the drug was not detectable after 3.3 h.
	Excised rabbit cornea for in-vitro drug release; rabbits for pharmacokinetics study [86]	Ibuprofen-loaded cationic liposomal eye drops	Ibuprofen entrapment rate in cationic liposomes was around 73% with 30 days. The cumulative release quantity was 1.64 times higher than that of conventional eye drops.
Flurbiprofen	Rabbit ocular surface inflammation model [80]	PLGA-NPs via topical eye drops	High drug entrapment efficiency (>90%) with 6-months storage stability. The developed NPs showed no sign of toxicity of irritation on ocular tissues. Good anti-inflammatory efficacy with controlled and continuous drug delivery.
	In-vitro corneal penetration test in isolated rabbit corneas; In-vivo ocular irritation and pre-corneal retention studies in rabbits [31]	Topical chitosan-coated liposomal formulation	Encapsulating drug in chitosan-coated liposomes reduced drug-associated ocular irritation, increased entrapment efficiency, and extended transport time across the cornea. The AUC_0–10min_ showed 2.84- and 1.53-fold higher than that of conventional eye drop and liposomal formulation without chitosan, respectively.
	In-vitro ocular irritation test; In-vivo experiments in rabbits [85]	Ultrasound-engineered NLCs via single instillation	Ocular tolerance assessment showed physico-chemical stability with high ocular tolerance.
Flurbiprofen axetil	Rabbit endotoxin-induced uveitis models [87]	Topical nanoemulsions eye drops	Aqueous humor pharmacokinetics showed the optimised emulsion formulations contain castor oil to tween 80 wt% ratio of 0.5:0.4. The optimised 0.1% flurbiprofen axetil-emulsion formulation showed better anti-inflammation effect relative to 0.03% flurbiprofen sodium ophthalmic solution.
Indomethacin	Alkaline-burned rabbits [81]	Chitosan-coated nanoemulsion eye drops	Fast release during the first hour followed by slow gradual drug release of 86% during 24 h. Therapeutic concentration was achieved in the cornea. Reduced corneal ulceration with no corneal cellular infiltration with indomethacin nanoemulsion treatment.
Lornoxicam	In-vivo ocular irritation test in rabbits [28]	Polymeric nanomicelles eye drops	The optimized loroxicam-loaded nanomicelles formulation showed 73-fold increase of solubility with about 60% drug release within 6 h. No physical irritation on rabbits’ eyes but the histopathological results revealed subacute inflammation after repeated instillation. ^1^HNMR analysis revealed complete encapsulation of drug.
